# GK-11: a Novel Cationic Peptide with Antibiofilm Activity against *Staphylococcus aureus* and *Pseudomonas aeruginosa*

**DOI:** 10.1007/s12602-026-10963-6

**Published:** 2026-03-03

**Authors:** Simay Aldağ, Güler Tuba Buğdacı, Şeymanur Çobanoğlu, Erdem Erkengez, Mehmet Enes Arslan, Abdurrahim Kadı, Serkan Örtücü, Mesut Taşkın, Ayşenur Yazıcı

**Affiliations:** 1 Faculty of Science, Molecular Biology and Genetics Department, Erzurum Technical University, Erzurum, Turkey; 2 Molecular Microbiology Laboratory, Erzurum Technical University, High Technology Research and Application Centre (YUTAM), Erzurum, Turkey; 3 Faculty of Science, Molecular Biology and Genetics Department, Atatürk University, Erzurum, Turkey

**Keywords:** Antibiofilm peptide, GK-11, Staphylococcus aureus, Pseudomonas aeruginosa, Caenorhabditis elegans

## Abstract

Biofilms are implicated in most chronic infections and exhibit up to 1000-fold higher antibiotic resistance than planktonic cells, creating an urgent need for new antibiofilm agents. Here, we characterized GK-11, an 11-amino acid derivative of pleurocidin. Although GK-11 showed limited antimicrobial activity (MIC: 64 µg/mL for *Staphylococcus aureus* and 256 µg/mL for *Pseudomonas aeruginosa*), it demonstrated potent antibiofilm effects at sub-MIC levels (MBIC: 32 µg/mL and 16 µg/mL, respectively). Microscopy and SEM confirmed disruption of biofilm structure, while qRT-PCR revealed downregulation of key virulence genes. GK-11 was non-toxic to *Caenorhabditis elegans* and maintained > 80% viability in human fibroblasts. In vivo, GK-11 improved worm survival against MRSA and PAO1 infection. These results identify GK-11 as a promising, low-toxicity peptide with selective antibiofilm activity for potential therapeutic development.

**Supplementary Information:**

The online version contains supplementary material available at 10.1007/s12602-026-10963-6.

## Introduction

Biofilms are clusters of microorganisms that produce extracellular polymeric substances (EPS). These substances consist of proteins, polysaccharides, lipids, DNA, and RNA, all of which play a critical role in the pathogenicity of biofilms [1, 2]. The EPS matrix not only supports microbial growth but also serves as a protective barrier. This structure hinders phagocytosis and restricts the access of immune system components, making biofilms particularly difficult for the host to eliminate [3]. Biofilm formation plays a central role in microbial pathogenesis. Biofilms associated with wound infections, medical devices, and chronic diseases complicate treatment and lead to increased healthcare costs. Bacteria such as *Pseudomonas aeruginosa* and *Staphylococcus aureus*, along with fungal species like *Candida albicans*, are among the primary contributors to clinical problems due to their ability to form biofilms, which significantly enhance their resistance to antibiotics and antifungal agents [4, 5].

In modern healthcare settings, biofilm-associated infections pose a major challenge. Biofilms exhibit remarkable resistance to both host immune defenses and antibiotic therapy, making them notoriously difficult to eradicate [6–8]. Studies have shown that bacteria within biofilms can be 10 to 1,000 times more resistant to antibiotics than their planktonic counterparts, further complicating treatment efforts [9]. Given that biofilms are estimated to be involved in approximately 80% of all infections, there is an urgent need for effective antibiofilm agents to combat their persistence and reduce their impact on public health [10].

Antimicrobial peptides (AMPs), also known as host defense peptides, represent the first line of defense against pathogens [11]. AMPs are evolutionarily conserved molecules found in a wide range of organisms from bacteria to humans, and are considered some of the earliest weapons of the immune system [11, 12]. They are typically small, cationic, and amphipathic peptides, rich in lysine and arginine, and exhibit broad-spectrum antimicrobial activity against bacteria, fungi, and viruses. In addition to their antimicrobial properties, AMPs also possess antibiofilm, immunomodulatory, anticancer, anti-inflammatory, wound-healing, and antioxidant activities. To date, nearly 5000 AMPs have been identified and are cataloged in the APD3 database [12, 13]. Although the concept of AMPs dates back to the early 20th century, their significance has increased dramatically in recent years due to the global rise in antibiotic resistance. Antibiofilm peptides (ABPs), a subgroup of AMPs, have been shown to inhibit biofilm formation or disrupt mature biofilms. Despite the serious clinical problems caused by biofilm-associated infections, no selectively approved antibiofilm drugs are currently available. Therefore, ABPs have emerged as promising candidates for the development of effective antibiofilm therapeutics [14, 15].

In the present study, we report the GK-11, a novel ABP candidate derived from pleurocidin. Pleurocidin was selected as a template listed in the APD3 database. It is a 25-residue peptide with broad-spectrum antimicrobial activity against both Gram-negative and Gram-positive bacteria, originally identified in the skin secretions of *Pleuronectes americanus* [16]. Here, we introduce GK-11, an 11-amino-acid derivative of pleurocidin, as a potential ABP. Using a range of assays, including broth microdilution, crystal violet staining, scanning electron microscopy, fluorescence microscopy, cytotoxicity assays, and a *Caenorhabditis elegans* infection model, we evaluated the antibiofilm activity of GK-11. Our results suggest that GK-11 is a promising candidate for development as an antibiofilm agent.

## Materials and Methods

### Materials and Bacterial Strains

All reagents were purchased from Sigma-Aldrich (USA) or Isolab, and all culture media were obtained from Neogen (UK), unless otherwise stated. The antimicrobial and antibiofilm activities of the GK-11 peptide were evaluated using *Pseudomonas aeruginosa* (PAO1) and Methicillin-resistant *Staphylococcus aureus* (MRSA, ATCC 43300). Bacterial strains were cultured on Mueller-Hinton agar (MHA) plates and incubated overnight at 37 °C under aerobic conditions to obtain actively growing colonies [17].

### Synthesis and Purification of GK-11

The GK-11 peptide was chemically synthesized by GenScript (Piscataway, NJ, USA). The purity and molecular weight of the peptides were confirmed by GenScript using analytical high-performance liquid chromatography (HPLC) and mass spectrometry (MS) (Figures S1 and S2).

Peptide stock solutions were prepared by dissolving the lyophilized peptide powder in sterile distilled water (dH₂O) and stored at −20 °C. Fresh working solutions were prepared from stock immediately prior to each experiment [18].

### Antimicrobial Activity

#### Microdilution Assay

The minimum inhibitory concentration (MIC) of GK-11 was determined using the broth microdilution method according to Clinical and Laboratory Standards Institute guidelines [19]. Briefly, 100 µL of bacterial suspension adjusted to 0.5 McFarland standard (approximately 1 × 10⁸ CFU/mL) was added to each well of a 96-well plate. An equal volume (100 µL) of sterile Mueller-Hinton Broth (MHB) containing serial two-fold dilutions of GK-11 (ranging from 0 to 256 µg/mL) was added to each well. After 24 h of incubation at 37 °C, MIC was defined as the lowest peptide concentration at which no visible bacterial growth was observed. Ampicillin, tobramycin, and ciprofloxacin were used as positive controls.

To determine minimum bactericidal concentration (MBC), aliquots (10 µL) from wells showing no visible growth, corresponding to the MIC and higher concentrations, were aseptically plated onto MHA medium and incubated at 37 °C for 18–24 h. The MBC was defined as the lowest peptide concentration that yielded no detectable colony formation on agar [20].

#### Biofilm Formation Assay

The antibiofilm activity of GK-11 was evaluated using a crystal violet (CV) assay [21]. Bacterial suspensions adjusted to 0.5 McFarland were inoculated (100 µL/well) into sterile 96-well polystyrene microplates. GK-11 was then added at final concentrations ranging from 0 to 256 µg/mL in a total volume of 200 µL. Plates were incubated statically at 37 °C for 48 h. Wells were subsequently washed three times with PBS to remove non-adherent cells. The attached biofilms were stained with 0.1% CV for 20 min. Excess dye was removed with tap water, and the stained biofilms were solubilized with 30% acetic acid. Absorbance was measured at 595 nm using a microplate reader. The lowest peptide concentration that completely inhibited biofilm formation was recorded as the minimum biofilm inhibitory concentration (MBIC).

#### Biofilm Eradication Assay

To assess the ability of GK-11 to eradicate mature biofilms, bacterial suspensions (100 µL at 0.5 McFarland) were seeded into 96-well plates and incubated statically at 37 °C for 24 h to allow biofilm formation. Wells were then washed three times with PBS to remove planktonic cells. Serial concentrations of GK-11 (0–256 µg/mL) were added and plates were incubated for 1 h at 37 °C. After treatment, wells were washed with PBS, stained with 0.1% CV for 20 min, and treated with 30% acetic acid. Biofilm biomass was quantified by measuring absorbance at 595 nm [21].

#### Dual-species Biofilms

For dual-species biofilms, *P*,* aeruginosa* and *S. aureus* were used. Overnight cultures of each strain were grown separately in tryptic soy broth (TSB) supplemented with 1% glucose and 2.5% NaCl. The bacterial cells were harvested by centrifugation at 6000 rpm for 10 min, washed, and resuspended in the same medium. Optical densities were adjusted to OD₆₀₀ = 0.1, and the two suspensions were mixed at a 1:1 ratio to initiate dual-species biofilm formation [22]. Using these cultures and the protocols described above, the effects of GK-11 on biofilm formation and eradication were investigated.

#### Cell Proliferation and Viability Assays

CVDK-8 cell viability test kit (Cat no: CVDK-8, Eco-Tech, Turkey) was used to determine cell proliferation according to kit protocols. For this analysis, 96-well plates were seeded with 0.5 McFarland concentration of bacterial cells per well. Then, the cells were incubated for 2 h with GK-11 (4xMIC). After that, bacteria were incubated with 10 µl of CVDK-8 solution for 4 h. Finally, spectrophotometric measurements at a wavelength of 450 nm were taken (Multiskan GO, Thermo Scientific).

The viability of *P. aeruginosa* and *S. aureus* after exposure to GK-11 (4xMIC) was determined by the cell-check viability/cytotoxicity kit (ABP Biosciences Inc.) for bacterial cells [23]. Briefly, *P. aeruginosa* and *S. aureus* were incubated at 37 °C for 16 h, centrifuged at 1000×g for 5 min and resuspended in 1 mL of 0.85% NaCl solution. Then, GK-11 was applied to the samples. Afterwards, fluorescent dyes NucView Green (1,5 µL) and propidium iodide (1,5 µL) were added to the bacterial suspension, followed by incubation at room temperature for 15 min. The control and treated samples were placed on the slides and detected under fluorescence microscopy (Zeiss, Axio Scope A1).

#### Scanning Electron Microscopy Analysis

SEM (Zeiss, Sigma 300) analysis was performed to determine the effect of GK-11 on bacteria *S. aureus* and *P. aeruginosa*. Firstly, bacterial cells were grown on sterile discs placed in plates at 37 °C for 48 h, with 1/2 MIC, MIC and control groups. Upon completion of the incubation period, unattached cells were eliminated through washing procedures. Subsequently, the entrapped cells underwent fixation with 5% glutaraldehyde for 15 min, followed by incubation in ethanol concentrations of 20%, 50%, 80%, and 100% for 10 min. The image was taken after the gold coating was applied [24].

### RT-PCR Analysis to Determine Selective Virulence Gene Expression

#### RNA Isolation and cDNA Synthesis

Total RNA from bacteria treated with GK-11 at a concentration of 1/2 MIC was isolated using a TRIzol RNA extraction kit (Invitrogen™, TRIzol™ Reagent) according to the manufacturer’s protocol. The obtained RNA was equalized to 1 µg concentration. cDNA was then synthesized using a commercial kit (Applied Biosystems™ High Capacity cDNA Reverse Transcription Kit).

#### qRT-PCR

Expressions of selected virulence genes (*P. aeruginosa*: *lasB*,* pilA*,* algD*,* oprI*; *S. aureus*: *hla*,* agrA*,* blaZ*,* pbp2a*) were analyzed with the rotor gene RT-PCR system using HOT FIREPol^®^ EvaGreen^®^ qPCR Supermix (Solis BioDyne, Estonia). The list of primers is indicated in Table 1. In qRT-PCR, transcript levels were normalized to *rhoD* and *gyrB* genes for *P. aeruginosa* and *S. aureus*, respectively. The expression level was analyzed using the 2^-(ΔΔCt)^ method [25].

**Table 1:** Primers used in qRT-PCR analysis

Genes	Primer Sequences	References
***gyrB***	F: 5’ ATCTGGTCGTGACTCTAGAA3’ R: 5’ TGTACCAAATGCTGTGATCA3’	Bezar vd, 2019
***hla***	F: 5’GTACAGTTGCAACTACCTGA3’ R: 5’CCGCCAATTTTTCCTGTATC3’	Bezar vd, 2019
***agrA***	F: 5’ACGAGTCACAGTGAACTTAC3’ R: 5’GACAACAATTGTAAGCGTGT3’	Bezar vd, 2019
***blaZ***	F: 5’TGCTTTAAATACTAAAAGTGGTAAGG3’ R:5’AGCAACTATATCATCTTTGTTAATATG3’	Alexander vd, 2020
***pbp2a***	F: 5’ACTTAAAACAAGCAATAGAATCATCAG3’ R: 5’AATTTGAGCATTATAAAATGGATAATCAC3’	Alexander vd, 2020
***rpoD***	F: 5’CATCCGCATGATCAACGACA3’ R: 5’GATCGATATAGCCGCTGAGG3’	Beaudoin et al. 2012
***lasB***	F: 5’GGAATGAACGAAGCGTTCTCCGAC3’ R: 5’TTGGCGTCGACGAACACCTCG3’	Faraji vd, 2016
***pilA***	F: 5’ CAGAGGCGACTGGTGAAATC3’ R: 5’ AGGGTAGAGTCAGCCGGAAT3’	Panayidou vd, 2020
***algD***	F: 5’GCGACCTGGACCTGGGCT3’ R: 5’TCCTCGATCAGCGGGATC3’	Beatrice vd, 2004
***oprI***	F: 5’ AACAGCGGTGCCGTTGAC3’ R: 5 GTCGGAGCTGTCGTACTCGAA3’	Beatrice vd, 2004

### Cytotoxicity Detection Assays

#### Cell Culture Conditions

Human dermal fibroblasts cell line (HDFa, ATCC^®^ PCS-201-012) was cultured utilizing Dulbecco’s Modified Eagle Medium (DMEM, Gibco^®^) as the basal nutrient medium. To enrich the medium, fetal bovine serum (FBS, acquired from Sigma-Aldrich^®^) was added at a concentration of 10%, alongside a 1% solution of penicillin/streptomycin (Sigma-Aldrich^®^) to prevent bacterial contamination. The cells were incubated under a precisely controlled atmosphere, maintained at a constant temperature of 37 °C and enriched with 5% CO_2_. This environment was sustained until the cell cultures achieved a confluence ranging from 80% to 90%, indicating optimal density for further processing. Upon reaching the desired confluence, the HDFa cells were detached using a trypsin/EDTA solution (Sigma-Aldrich^®^), facilitating their collection for downstream applications. Following detachment, the cells were transferred and evenly distributed into 48-well plates, preparing them for the subsequent experimental assays [26].

#### MTT and LDH assays

The cytotoxic effects of GK-11 on HDFa cell lines were investigated through commercial cell viability kits (MTT Cell Growth Assay Kit, Sigma-Aldrich^®^ CT01 and Bio-Vision LDH assay kit, LDH; cat.no ab65393, Abcam). Initially, cells were plated in 96-well plates at densities conducive to optimal interaction with the GK-11. These cells were then treated with a gradient of peptide concentrations ranging from 0.25 to 256 µg/ml and incubated for a duration of 24 h to allow for sufficient exposure. To quantify cell viability post-treatment, the 3-(4, 5-dimethylthiazol-2-yl)−2, 5-diphenyl tetrazolium bromide (MTT) and Lactate Dehydrogenase (LDH) assays were employed. MTT assay involved the addition of 10 µL of MTT reagent (concentration of 5 mg/ml) to each of the wells, followed by an incubation period at 37 °C for 3 h, facilitating the conversion of MTT to formazan by metabolically active cells. Subsequently, to solubilize the formazan crystals, 100 µL of dimethyl sulfoxide (DMSO, Sigma-Aldrich^®^) was introduced to each well. The absorbance of the resulting solution was measured at a wavelength of 570 nm using a BioTek^®^ microplate reader. The LDH assay involved transferring 100 µL of supernatant from experimental samples into a new well of a 96-well plate, followed by adding 100 µL of the reaction mixture from the Bio-Vision LDH assay kit. The mixture was incubated at room temperature for 30 min. Post-incubation, absorbance at 450 nm was measured using BioTek^®^ microplate reader to quantify LDH release, indicating cell membrane damage. Results were analyzed by comparing with control values as per the kit’s instructions. Cells treated with medium containing 1% Triton X-100 were used as the positive control, while untreated cells served as the negative control [26].

### *Caenorhabditis elegans* Toxicity and Infection Assay

The *C. elegans* N2 wild-type strain was used to assess both the toxicity and in vivo efficacy of GK-11. The nematodes were maintained under standard laboratory conditions on nematode growth medium (NGM) agar plates at 25 °C and fed with *Escherichia coli* OP50 as a food source [27].

Slow killing assays were conducted on NGM plates supplemented with 5-fluoro-2′-deoxyuridine (FUDR) to prevent progeny development. For each assay, approximately 20 young adult worms were transferred to assay plates and exposed to GK-11 at ½xMIC concentration. The plates were incubated at 25 °C, and nematode viability was monitored daily. Worms were scored as dead if they showed no movement in response to gentle physical stimulation with a pipette tip [27, 28]. All assays were performed in triplicate.

### Statistical Analyses

All experiments were repeated at least three times. The analysis was performed using One-way ANOVA and Student’s *t*-test with GraphPad Prism 8 or OriginPro 9. Statistical significance was determined using a p-value threshold of 0.05.

## Results

### Antimicrobial and Antibiofilm Activity of GK-11

The antimicrobial activity was determined against *P. aeruginosa* and *S. aureus* bacterial strains (Table 2). GK-11 demonstrated limited bactericidal and bacteriostatic activity, particularly against *P. aeruginosa*. For S. aureus, the MIC was determined to be 64 µg/mL. As positive controls, the MIC values of ampicillin, tobramycin, and ciprofloxacin against *P. aeruginosa* were determined as > 256, 4, and 2 µg/mL, respectively. For *S. aureus*, the corresponding MIC values of these antibiotics were > 128, 16, and 32 µg/mL, respectively. The MBC value of GK-11 was determined to exceed 256 µg/mL.

**Table 2:** Antimicrobial and antibiofilm activity of GK-11 against *P. aeruginosa* and *S. aureus*

Bacterial strain	MIC (µg/mL)	MBC (µg/mL)	MBIC (µg/mL)
*P. aeruginosa* PAO1	256	> 256	16
*S. aureus* ATCC 43,300	64	> 256	32

**Fig. 1:**
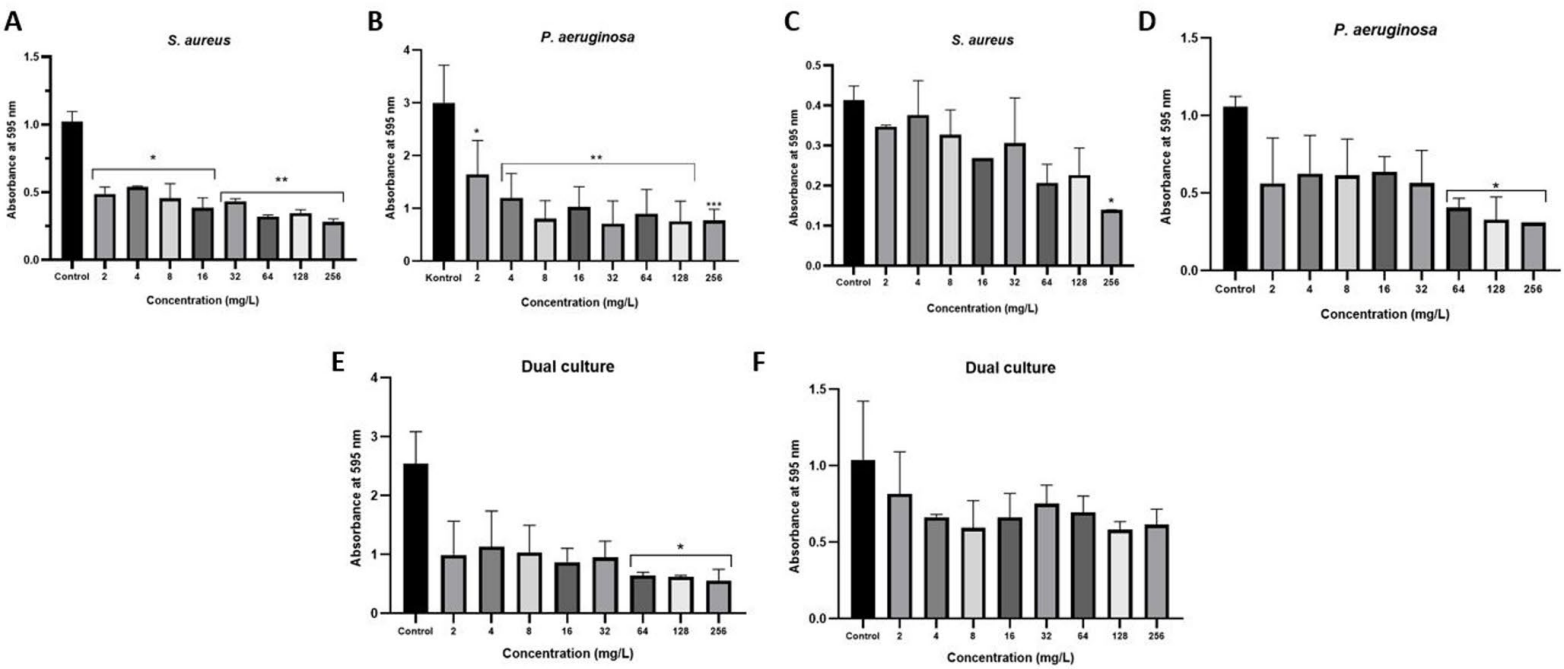
Effects of the GK-11 on biofilm formation and eradication in *S. aureus*, *P. aeruginosa*, and dual-species cultures. Biofilm biomass was quantified using the crystal violet assay and measured as absorbance at 595 nm after treatment with increasing concentrations (0–256 mg/L). **A**: Inhibition of biofilm formation in *S. aureus*. **B**: Inhibition of biofilm formation in *P. aeruginosa*. **C**: Eradication of pre-formed *S. aureus* biofilms. **D**: Eradication of pre-formed *P. aeruginosa* biofilms. **E**: Inhibition of biofilm formation in dual-species biofilms. **F**: Eradication of pre-formed dual-species biofilms. Data are presented as mean ± SD of at least three independent experiments. Statistical significance was determined by comparison with the untreated control (*=*p* < 0.05, **=*p* < 0.01, ***=*p* < 0.001)

In contrast, CV assays revealed that GK-11 possessed a considerably higher antibiofilm activity compared to its antibacterial activity (Fig. 1A and B). The MBIC was markedly lower than the MIC for both pathogens (Table 2), indicating a strong biofilm-targeting potential independent of bactericidal effects.

In addition to inhibiting biofilm formation, GK-11 exhibited a concentration-dependent capacity to eradicate established biofilms. Pre-existing biofilms of *S. aureus* were disrupted at 256 µg/mL (Fig. 1C), whereas a significant reduction in *P. aeruginosa* biofilms was observed from 64 µg/mL onward (Fig. 1D).

The efficacy of GK-11 was also evaluated against dual-species biofilms formed by *S. aureus* and *P. aeruginosa*. GK-11 significantly inhibited biofilm formation in dual cultures from 64 µg/mL onward (Fig. 1E); however, it did not exhibit eradication activity against established dual-species biofilms (Fig. 1F).

### Proliferation and Cell Viability of *P. aeruginosa* and *S. aureus*

**Fig. 2:**
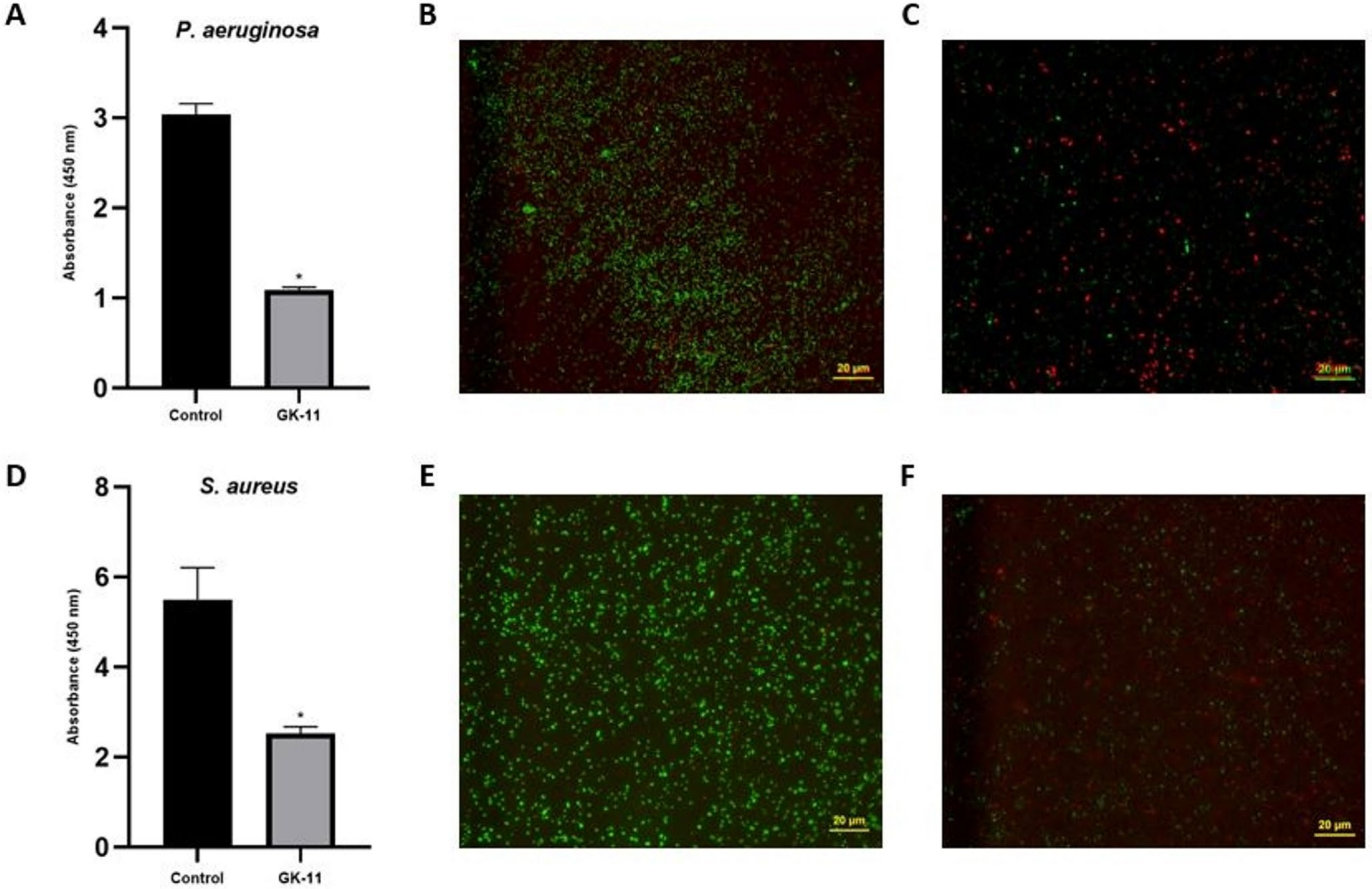
Cell proliferation and viability of *P. aeruginosa* and *S. aureus* after GK-11 treatment. **A**,** D**: Cell proliferation of *P. aeruginosa* and *S. aureus*, respectively, using CVDK-8 assay. **B**,** E**: Overlay images of live (green fluorescent) and dead (red fluorescent) control of *P. aeruginosa* and *S. aureus*, respectively, using fluorescence microscope. **C.** Overlay images of live (green fluorescent) and dead (red fluorescent) *P. aeruginosa* treated with GK-11 are shown. **F.** Overlay images of live (green fluorescent) and dead (red fluorescent) *S.aureus* treated with GK-11 are shown

CVDK-8 assays demonstrated that GK-11 treatment (4×MIC for 2 h) reduced the metabolic activity of planktonic *P. aeruginosa* and *S. aureus* cells by 35.85% and 45.98%, respectively (Fig. 2A and D).

In fluorescence imaging, untreated control cells (Fig. 2B and E) exhibited strong green fluorescence, indicative of viable populations. GK-11-treated *P. aeruginosa* cells displayed mixed green and red fluorescence (Fig. 2C), signifying partial cell death. Similarly, *S. aureus* cells exposed to GK-11 (4×MIC) showed dual staining patterns (Fig. 2F), indicating membrane compromise and reduced viability.

### SEM Analysis

**Fig. 3:**
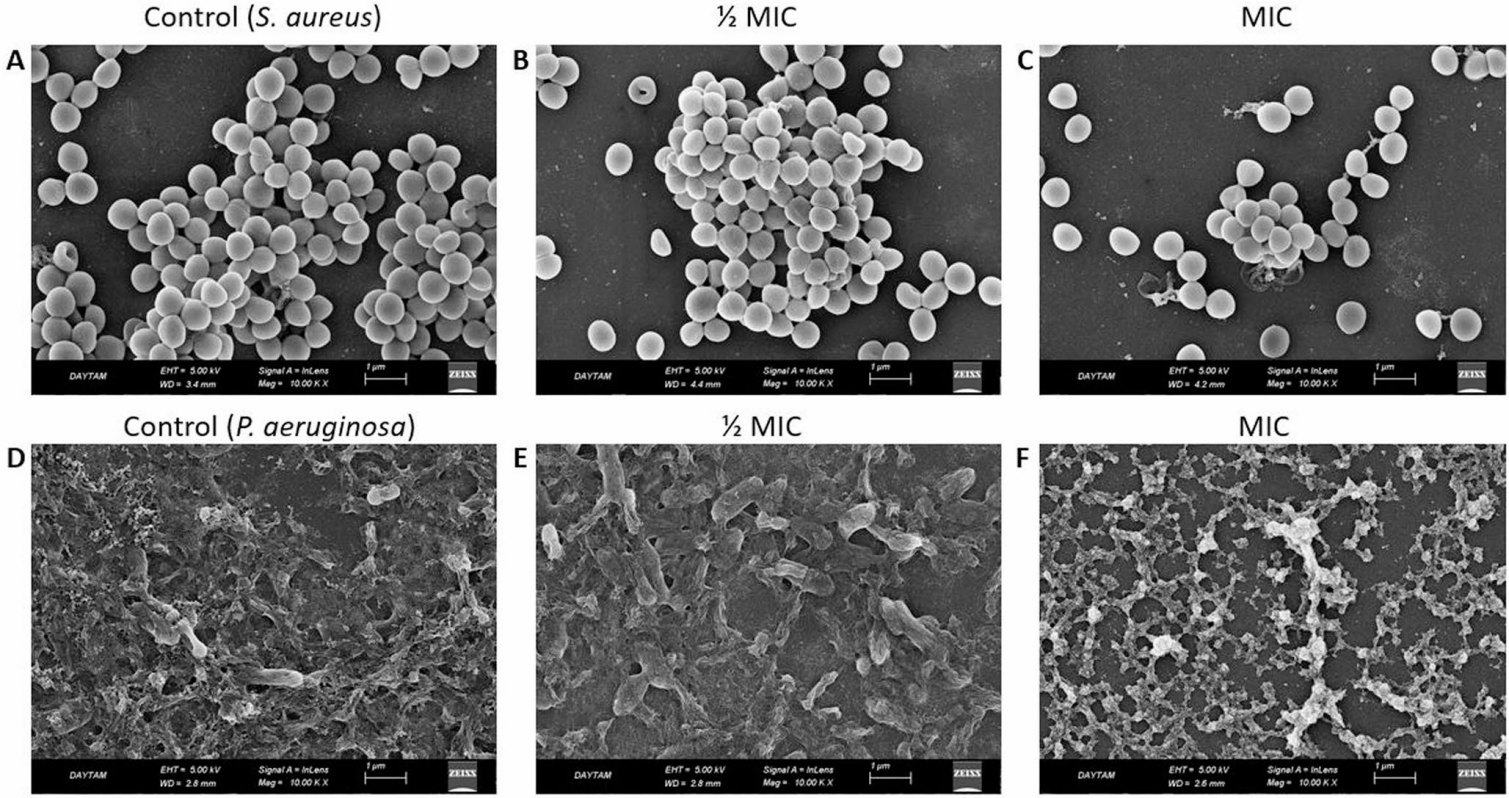
Scanning electron micrographs of *S. aureus* and *P. aeruginosa* biofilm in the presence GK-11 (MIC and 1/2 MIC). **A**,** D**: SEM images of *S. aureus* and *P. aeruginosa* biofilm structure as a control, respectively. **B**,** E**: Treatment with 1/2 MIC value of GK-11. **C**,** F**: MIC value of of GK-11 for *S. aureus* and *P. aeruginosa*, respectively

To further investigate the impact of GK-11 on biofilm architecture and bacterial surface adherence, SEM imaging was performed. As shown in Fig. 3, untreated control groups of *P. aeruginosa* and *S. aureus* formed dense, well-structured biofilms. In contrast, a reduction in surface-adhering cells was observed in bacterial cultures treated with GK-11 at both ½ MIC and MIC concentrations, confirming the peptide’s strong antibiofilm activity.

### Effect of GK-11 on the Expression Level of Selected Virulence Genes

**Fig. 4:**
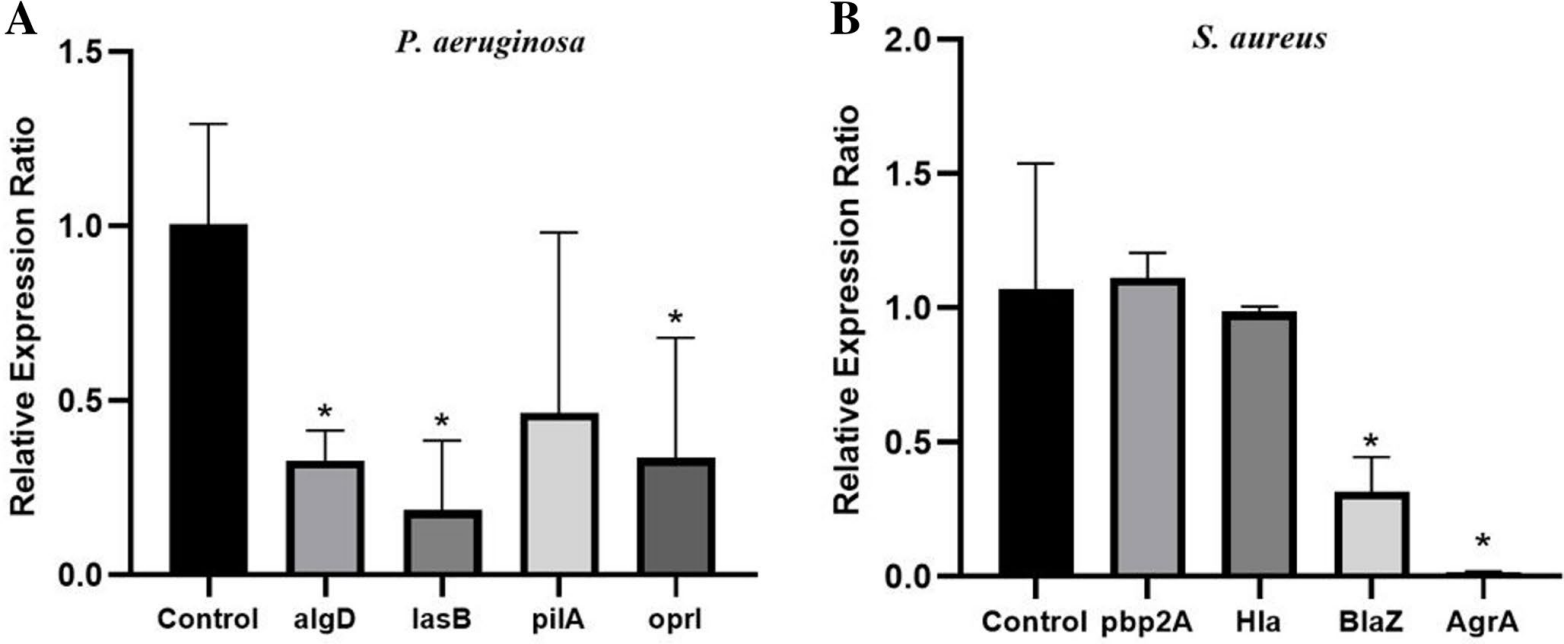
Relative expression of selected virulence genes with control and GK-11 treatment. Virulence genes levels were normalized with *rpoD* and *gyrB* genes for **(A)** *P. aeruginosa* and **(B)** *S. aureus*, respectively (*=*p* < 0.05)

The relative expression levels of the selected virulence genes were evaluated at 16 h after treatment of GK-11. The mRNA levels of virulence genes were compared with the control group in which GK-11 was not treated. The relative expression levels of virulence genes are presented in Fig. 4. As shown in Fig. 4A, expression of *algD*,* lasB* and *oprL* genes of *P. aeruginosa* was downregulated within the 16 h treatment of GK-11 compared with the control culture. However, there was no significant change in the *pilA* gene compared to the control. As shown in Fig. 4B, expression of *blaZ* and *agrA* genes of *S. aureus* was downregulated within the overnight treatment of GK-11 compared with the control culture. However, there was no significant change in the *pbp2A* and *hla* genes compared to the control.

### Cytotoxic effect of GK-11 on Human HDFa Cells

**Fig. 5:**
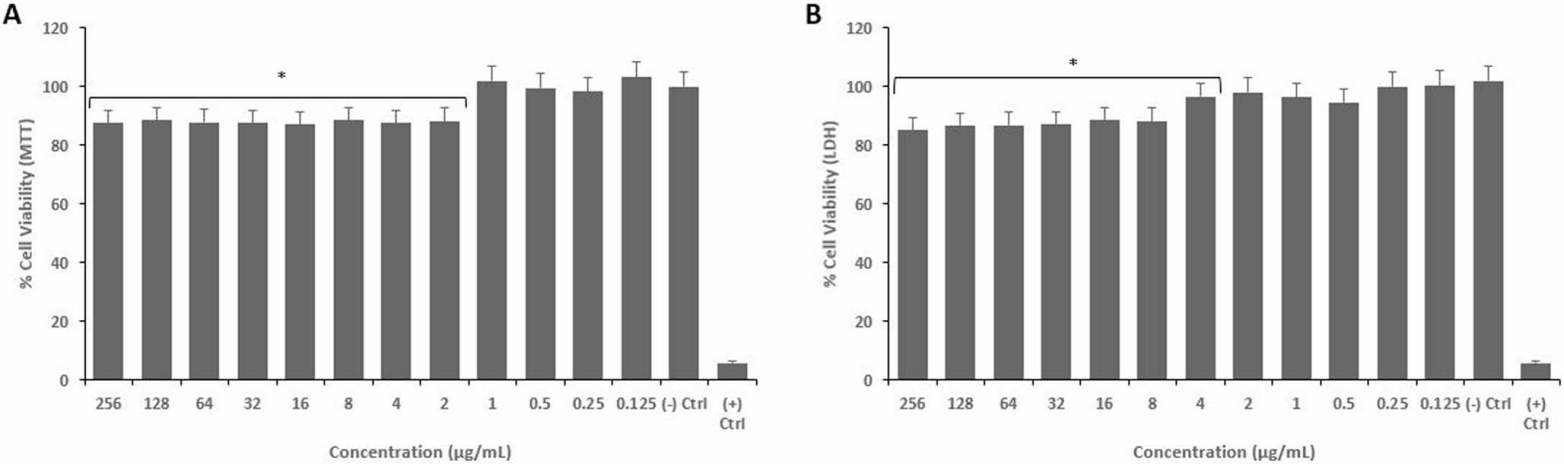
**(A)** MTT and **(B)** LDH cell viability analyses of GK-11 on the HDFa cell line for 24 h of applications (*=*p* < 0.05)

MTT cell viability and LDH cytotoxicity assays were employed to assess the effects of GK-11 on HDFa cell cultures. These assays were integral in determining whether the GK-11 harbored any cytotoxic properties that could potentially compromise cell health or viability. Through the application of these assays, it was aimed to discern the safety profile of the GK-11, particularly at varying concentrations, to understand its cytotoxicity implications. The peptide exhibited a concentration-dependent effect on cell viability. Notably, even at a concentration of 256 µg/mL, it maintained over 80% cell viability. This effect was significantly distinct from the outcomes observed with the negative control, underscoring the unique biological activity of GK11 (Fig. 5A and B).

### Effects of GK-11 on the Survival of *C. elegans*

**Fig. 6:**
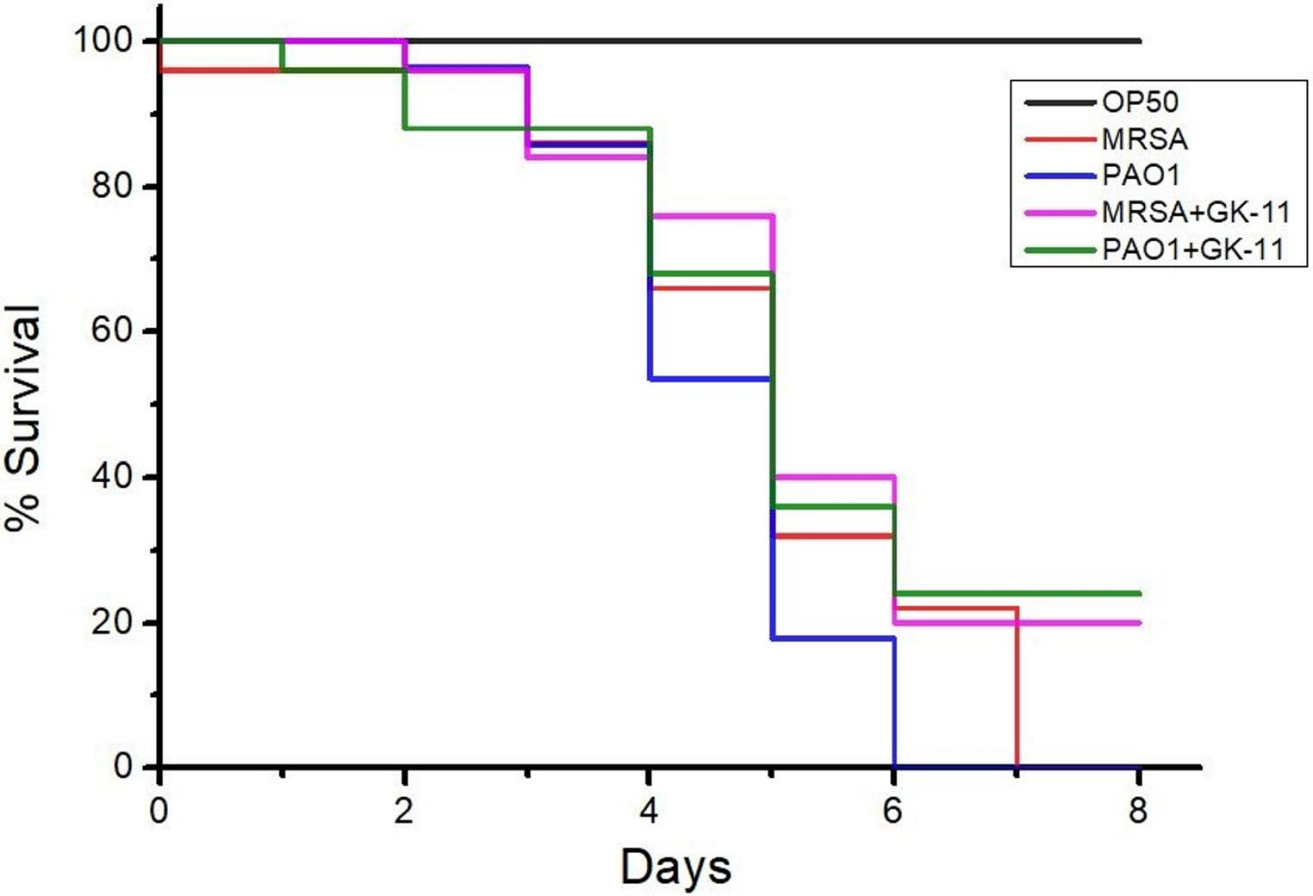
*C. elegans* lifespan and killing assays of PAO1 and MRSA

GK-11 by themselves did not display any toxic activity against *C. elegans*. To test the ability of GK-11 to protect against infections, we utilised *C. elegans* wild type. Prior research has established that *P. aeruginosa* PAO1 and *S. aureus* exhibit lethality toward *C.elegans* when cultured on brain heart infusion (BHI) agar. In the slow-killing assay, untreated controls infected with *P. aeruginosa* PAO1 and *S. aureus* demonstrated 100% death after 6 and 7 days, respectively. However, treatment with GK-11 preserved 24% viability in PAO1-infected nematodes and 20% in *S. aureus*-infected nematodes by day 8 (Fig. 6). These results provide strong evidence that GK-11 reduce expression of pathogenic traits in *S. aureus* and PAO1.

## Discussion

Biofilm-mediated infections caused by MRSA and multidrug-resistant *P. aeruginosa* pose a critical threat to global public health. In recent decades, the alarming rise of antimicrobial resistance has escalated this issue further [29, 30]. While the previous estimates of the World Health Organization (WHO) suggested that 10 million people would lose their lives due to resistance-related reasons by 2050, a new study conducted in 2022 states that these estimated numbers will now be exceeded [31].

Biofilm formation is one of the primary mechanisms that facilitates bacterial resistance. Biofilms form complex, three-dimensional structures that hinder the penetration of antimicrobial agents and protect bacteria from host immune responses, thereby significantly reducing treatment efficacy [32]. Despite the clinical burden, the development of agents specifically targeting biofilm-related infections remains limited [33, 34]. AMPs have emerged as promising alternatives due to their unique modes of action, broad-spectrum antimicrobial properties, and ability to disrupt biofilms without inducing significant resistance [35].

In this study, we designed GK-11, a novel short peptide derived from Pleurocidin (Table 3). According to the APD3 database, pleurocidin (ID: AP00166), a 25-amino-acid cationic AMP derived from the skin secretions of *P. americanus*, exhibits MIC values in the range of 8 µM against *S. aureus* and 4–8 µM against *P. aeruginosa* strains (https://aps.unmc.edu/). MIC values reported by Cole et al. [36] ranged between 17.7 and 35.0 µg/mL for *S. aureus*, while values exceeding 35.0 µg/mL were observed for *P. aeruginosa*. Similarly, Lee and Lee [37] documented MIC values between 0.9 and 7.4 µM for *S. aureus* and between 0.9 and 3.7 µM for *P. aeruginosa*. Although these findings align generally with data reported in databases, discrepancies may be attributed to strain-specific susceptibility differences. In the current study, the MIC value of the novel peptide was determined to be 64 µg/mL for MRSA and 256 µg/mL for *P. aeruginosa*. Although its antimicrobial activity is lower than that of pleurocidin, the peptide exhibited significantly stronger antibiofilm activity. Notably, the MBIC of GK-11 was found to be only 32 µg/mL for *S. aureus* and 16 µg/mL for *P. aeruginosa*, values that are 2-fold and 16-fold lower than their respective MICs. This striking disparity indicates that GK-11 is markedly more effective at disrupting or preventing biofilm formation than at inhibiting planktonic bacterial growth. Such selective antibiofilm activity, particularly against pathogens with high clinical relevance like MRSA and *P. aeruginosa*, underscores the therapeutic potential of GK-11 as a biofilm-targeted agent rather than a broad-spectrum bactericide.

**Table 3:** Molecular weight, hydrophobic ratios and overall net charge of pleurocidin and GK-11

Sequence	Molecular Weight (kDa)	Hydrophobic Ratio	Net Charge
Pleurocidin	2.72	44%	+ 7
GK-11	1.36	45%	+ 4.25

Structurally, GK-11 differs substantially from its parent peptide pleurocidin (GWGSFFKKAAHVGKHVGKAALTHYL) by being an 11-residue truncated analogue (GKRVWKRALIH-NH₂) with a C-terminal amidation. This shortened sequence retains key cationic and hydrophobic residues essential for antimicrobial activity but eliminates non-critical regions, potentially enhancing biofilm penetration and reducing susceptibility to proteolytic degradation [38, 39]. These structural modifications may account for the lower concentration values observed during biofilm inhibition compared to planktonic growth and may also underlie the superior antibiofilm activity of GK-11 relative to the parent pleurocidin [40].

The cell proliferation and viability assays further support the biofilm-disrupting potential of GK-11. Quantitative metabolic activity measurements using the CVDK-8 assay revealed a significant reduction in the viability of both *P. aeruginosa* and *S. aureus* following exposure to GK-11 at 4×MIC concentrations in 1 h. This reduction in absorbance indicates a decline in bacterial metabolic activity, suggestive of membrane destabilization or functional compromise. These findings were corroborated by fluorescence microscopy, where untreated control groups predominantly exhibited green fluorescence, indicating viable cells. In contrast, GK-11-treated samples showed diminished green signal alongside increased red fluorescence, marking membrane-compromised or dead cells. This dual staining pattern confirms that GK-11 compromises bacterial membrane integrity, leading to partial cell death even in planktonic populations. However, considering the high MIC and MBC values, the observed effect may be attributed more to sub-lethal membrane perturbation rather than outright bactericidal activity [41, 42].

SEM analysis provided direct visual evidence of the antibiofilm activity of GK-11 against both *S. aureus* and *P. aeruginosa*. In the untreated controls, *S. aureus* cells (Fig. 3A) formed dense aggregates, while *P. aeruginosa* cells (Fig. 3D) established a compact, multilayered biofilm network with extensive surface coverage. Following treatment with GK-11 at ½ MIC, a pronounced reduction in biofilm biomass and surface adhesion was observed for both pathogens (Fig. 3B and E). Only sparse bacterial cells remained, and extracellular polymeric substances appeared markedly diminished. At MIC, the disruption was even more apparent (Fig. 3C and F), with *S. aureus* surfaces showing isolated or absent coccal cells, and *P. aeruginosa* biofilms displaying substantial structural collapse, leaving behind scattered debris rather than organized communities. These morphological changes confirm that GK-11 exhibits potent biofilm-inhibiting activity even at sub-MIC levels. The observed architectural disruption, in conjunction with the peptide’s low MBIC values, suggests that it may inhibit initial bacterial adhesion and compromise biofilm matrix stability [43, 44].

In addition to inhibiting biofilm formation, GK-11 also eradicated mature biofilms in a concentration-dependent manner. These findings are significant because mature biofilms are EPS matrix and altered metabolic states of the embedded bacteria. The ability of GK-11 to reduce established biofilm biomass albeit at higher concentrations than those required for MBIC suggests that the peptide can penetrate or destabilize the EPS matrix or compromise cell-cell and cell-surface interactions within the biofilm. This dual action, involving both inhibition of biofilm formation and eradication of mature biofilms, underscores GK-11’s therapeutic potential for managing persistent biofilm-associated infections, especially in pathogens such as MRSA and multidrug-resistant *P. aeruginosa* where conventional antibiotics often fail [45].

Recent insights into polymicrobial biofilms highlight that interspecies interactions profoundly enhance biofilm resilience through cooperative EPS production, metabolic cross-feeding, and collective stress responses, ultimately leading to increased tolerance against antimicrobial agents [46]. GK-11 exhibited significant activity against dual species biofilms at high concentrations. In contrast, dual species biofilm eradication was more limited in pre-formed dual species biofilms, suggesting that mature dual species biofilms exhibit higher tolerance to GK-11 treatment. This differential response between biofilm formation and destruction may be a characteristic of dual-species biofilms that can restrict peptide penetration and reduce antibiofilm efficacy.

The significant downregulation of key *P. aeruginosa* virulence genes (*algD*, *lasB*, and *oprL*) following GK-11 treatment provides important insight into the antibiofilm activity of this peptide. Although the twitching and Swarming motility assay revealed a marked reduction in type IV pilus–dependent motility (Figure S3 and S4), the expression level of *pilA* did not show a statistically significant change, suggesting that GK-11 may impair pilus associated motility through post-transcriptional regulation or indirect effects rather than direct transcriptional suppression. Among the affected genes, *algD* plays a pivotal role in alginate biosynthesis by encoding GDP-mannose 6-dehydrogenase, a key precursor of the alginate biopolymer. Alginate constitutes a major structural component of the biofilm extracellular matrix and is critically involved in biofilm stability, immune evasion, and protection against antimicrobial agents [47]. *LasB* is a well-characterized quorum sensing regulated virulence factor that contributes to tissue damage through the degradation of host extracellular matrix proteins, including elastin and collagen [48]. In addition, *LasB* disrupts host immune responses by targeting immunoglobulins, complement components, and antiproteases and has been implicated in the induction of IL-1β mediated inflammatory responses during pulmonary infections [49]. Accordingly, the suppression of *lasB* expression following GK-11 treatment suggests an interference with quorum sensing regulated pathogenic pathways, which may contribute to attenuated virulence and reduced inflammatory damage. Furthermore, the observed decrease in *oprL* expression further supports the antibiofilm and antivirulence potential of GK-11. *OprL* is an outer membrane lipoprotein that plays a critical role in maintaining cell envelope integrity and regulating membrane permeability [50]. Altered *oprL* expression has been associated with a compromised outer membrane barrier, potentially facilitating enhanced penetration of antimicrobial agents.

Following GK-11 treatment of *S. aureus*, the expression levels of *blaZ* and *agrA* were downregulated, whereas no significant changes were observed in the expression levels of *pbp2a* and *hla*. The *blaZ* gene encodes the penicillinase (β-lactamase) enzyme, which hydrolyzes the β-lactam ring of penicillin and confers penicillin resistance in *S. aureus*, particularly upon exposure to β-lactam antibiotics [51]. The *agrA* gene is a central regulator of the quorum sensing system, promoting the transcription of RNAII and RNAIII and thereby enhancing the production of exoproteins [52]. Thus, downregulation of both *blaZ* and *agrA*, suggesting a potential inhibitory effect of GK-11 on β-lactam resistance mechanisms and quorum sensing–regulated virulence pathways [53]. Overall there is noticeable difference in the expression of more than one virulence gene which proves that our peptide has an effect on the virulence of these two microorganisms.

Cytotoxicity assays (MTT and LDH) indicated that GK-11 exhibits dose-dependent effects on mammalian cells [54]. At high concentrations, cell viability decreased significantly and remained around 80%. This indicates that there were statistically significant differences when compared to the negative control and positive control. In addition, when the concentration decreased to 1 µg/mL and below, cell viability increased and reached levels similar to the negative control group. This trend indicates that the compound did not show a significant cytotoxic effect at lower concentrations and was not toxic below a certain threshold concentration. This observation is consistent with similar studies showing that the peptide can only show cytotoxic effects at high concentrations and protect cells at low concentrations. The fact that there was no decrease in cell viability below 50% at any of the tested concentrations indicates that this compound has relatively low toxicity in the tested concentration range and has the potential for safe use at lower doses [54–56].

The *C. elegans* infection model further validated the in vitro findings, demonstrating that GK-11 is non-toxic to the host organism while providing significant protection against bacterial challenge [57]. In the control group fed with the non-pathogenic *E. coli* OP50, worm survival remained at 100% throughout the experimental period, confirming the baseline viability of the model. In contrast, infection with *P. aeruginosa* PAO1 or MRSA resulted in rapid mortality, with complete death occurring by day 6 and day 7, respectively. Treatment with GK-11 markedly improved nematode survival in both infection models. By day 8, survival rates were approximately 24% in PAO1-infected worms and 20% in MRSA-infected worms receiving GK-11, compared to 0% survival in untreated infected controls. These results indicate that GK-11 can attenuate bacterial virulence in vivo, potentially through disruption of biofilm formation and suppression of key virulence factors, as observed in the gene expression assays. In addition, to evaluate the potential toxic effects of GK-11, the peptide was administered together with *E. coli* OP50 in the feeding medium of *C. elegans*. Survival was monitored over a 10-day period, during which no decrease in viability was observed. These findings indicate that GK-11 does not exert detectable toxic effects under the tested conditions [56].

In conclusion, GK-11, a novel pleurocidin-derived short peptide, demonstrated potent and selective antibiofilm activity against MRSA and *P. aeruginosa* at concentrations substantially lower than its MIC values. The peptide not only inhibited biofilm formation but also eradicated mature biofilms, disrupted extracellular matrix architecture, and downregulated key virulence genes, while exhibiting minimal cytotoxicity at therapeutically relevant doses. Furthermore, in vivo validation using the *C. elegans* model confirmed its safety and capacity to improve host survival during bacterial infection [27, 57]. These results highlight GK-11 as a promising candidate for combating persistent biofilm-associated infections. Further studies should focus on structural optimization to enhance its antimicrobial and antibiofilm potency, as well as on elucidating its mechanisms of action and stability under physiological conditions.

## Conclusion

GK-11, a novel pleurocidin-derived peptide, showed potent and selective antibiofilm activity against MRSA and *P. aeruginosa* at concentrations far below its MIC values. It inhibited biofilm formation, eradicated mature biofilms, and reduced virulence while exhibiting low cytotoxicity and in vivo safety. These results position GK-11 as a promising lead for biofilm-targeted antimicrobial development.

## Supplementary Information

Below is the link to the electronic supplementary material.

Supplementary Material 1

## Data Availability

Data will be made available on request.
