# Supplementary material for: GK-11: a Novel Cationic Peptide with Antibiofilm Activity against Staphylococcus aureus and Pseudomonas aeruginosa
Source: Probiotics Antimicrob Proteins. 2026 Mar 3;18(5):7370–81. doi: 10.1007/s12602-026-10963-6 (PMC13369647; doi:10.1007/s12602-026-10963-6)
Supplement: Supplementary file 1 — Supplementary Material 1 [file 12602_2026_10963_MOESM1_ESM.docx]

**Supplementary Data**

**GK-11: A Novel Cationic Peptide Targeting Biofilm Formation and Disruption in *Staphylococcus aureus* and *Pseudomonas aeruginosa***

Simay Aldağ^1,2^, Güler Tuba Buğdacı^1,2^, Ruhane Tosunoğlu^1,2^, Şeymanur Çobanoğlu^1,2^, Erdem Erkengez^1,2,3^, Mehmet Enes Arslan^1^, Serkan Örtücü^1,2^, Mesut Taşkın^3^, Ayşenur Yazıcı^1,2*^

^1^Erzurum Technical University, Faculty of Science, Molecular Biology and Genetics Department, Erzurum, Turkey.

^2^Erzurum Technical University, High Technology Research and Application Centre (YUTAM), Molecular Microbiology Laboratory, Erzurum, Turkey

^3^Atatürk University, Faculty of Science, Molecular Biology and Genetics Department, Erzurum, Turkey


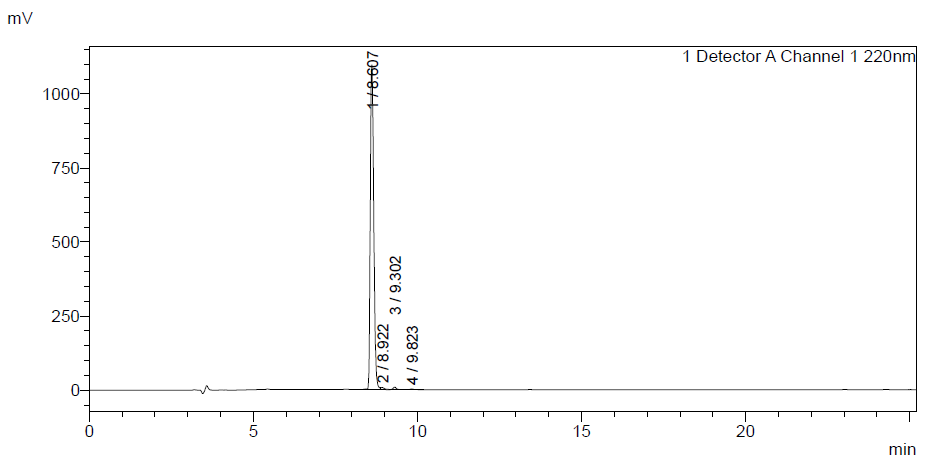


**Fig. S1:** High performance liquid chromatography of GK-11


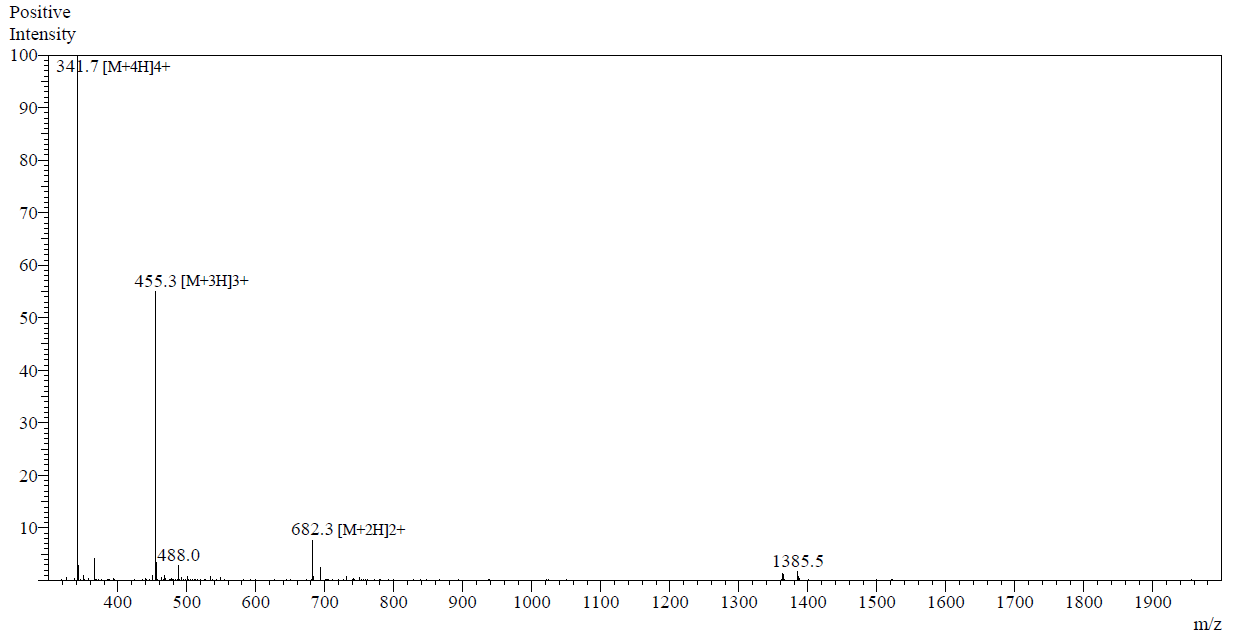


**Fig. S2:** Mass spectrometry of GK-11


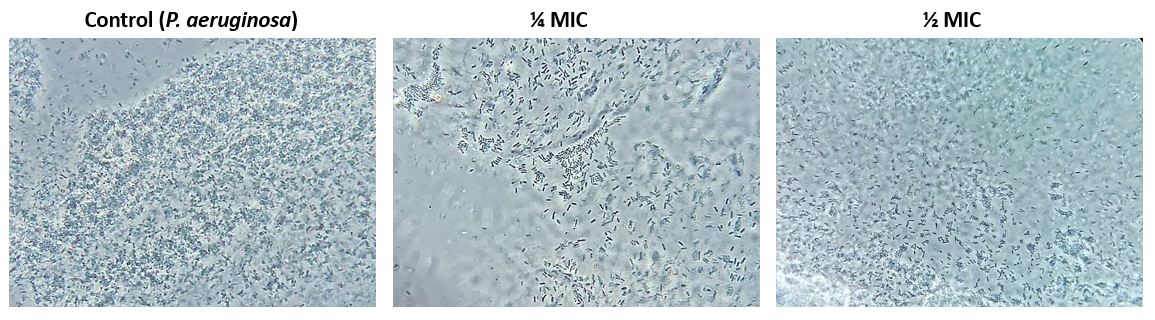


**Fig. S3:** Twiching Motility of *P. aeruginosa* (PAO1) treated with sub-MIC concentration of GK-11.


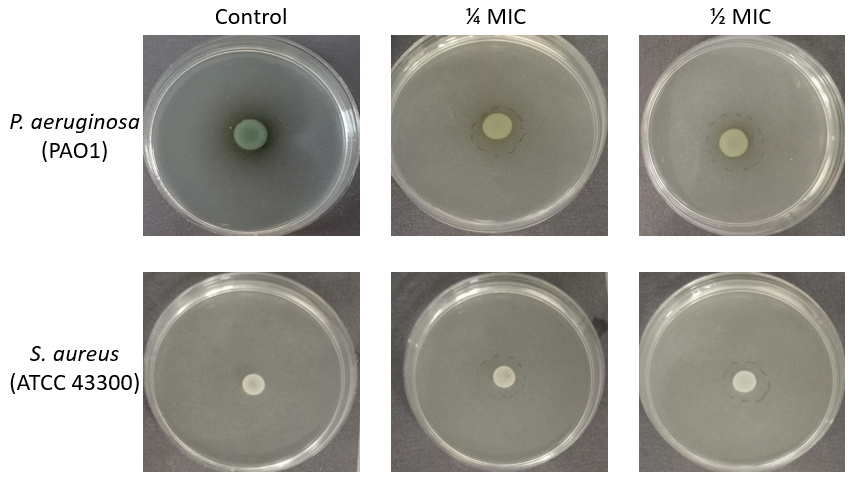


**Fig. S4:** Swarming Motility of *P. aeruginosa* (PAO1) and *S. aureus* (ATCC43300)
